# Repurposing an inhibitor of ribosomal biogenesis with broad anti-fungal activity

**DOI:** 10.1038/s41598-017-17147-x

**Published:** 2017-12-05

**Authors:** Nuo Sun, Dongmei Li, Yuhan Zhang, Kyle Killeen, William Groutas, Richard Calderone

**Affiliations:** 1 Georgetown University Medical Center, Washington DC, 20057 USA; 2 Wichita State University, Wichita, Kansas 67260 USA

## Abstract

The lack of new antifungal compounds with unique mechanisms of action is a concern for therapeutic management of patients. To identify inhibitors against human pathogenic fungi, we screened ~3000 compounds provided by the Developmental Therapeutics Program of NIH/NCI against a panel of pathogenic fungi including *Candida* species, *Aspergillus fumigatus*, and *Cryptococcus neoformans*. NSC319726 (a thiosemicarbazone) had broad antifungal activity in the range of 0.1–2.0 µg/ml and was also inhibitory to fluconazole-resistant isolates of *Candida* species. Synergy was demonstrated with NSC319726 and azoles, as well as caspofungin. The inhibitory concentration 50% (IC_50_) of NSC319726 was 35–800-fold higher than the Minimum Inhibitory Concentration 50% (MIC_50_ values), which indicates low compound toxicity to human cells *in vitro*. Transcriptome analysis of treated and untreated *C. albicans* using Gene Ontology (GO) revealed a large cluster of down regulated genes that encode translational proteins, especially those with ribosome biogenesis functions. As NSC319726 was first shown to have anti-cancer activity, its affects against human pathogenic fungi establish NSC319726 as a repurposed, off-patent compound that has potential antifungal activity. The minimal *in vitro* toxicity of lead optimized NSC319726 and its reasonable inhibitory activity against pathogens suggest advancing this compound to *in vivo* toxicity testing and protection studies against candidiasis.

## Introduction

Invasive fungal infections remain common globally, and mortality caused by these pathogens now is equal to or exceeds drug resistant-*Mycobacterium tuberculosis* and malaria^1^. In the case of blood-borne candidiasis and invasive aspergillosis, low sensitivity diagnostic tests and even drug resistance can be linked to poor patient outcome^2,3^. Patients are stratified by risk factors, and in the absence of positive blood cultures, may be treated empirically. *Candida* species, *Cryptococcus neoformans*, as well as *Aspergillus species* have been added recently to the Federal Drug Administration (FDA) list of pathogens that constitute public health threats in the USA (https://www.federalregister.gov/documents/2014/06/05/2014-13023/establishing-a-list-of-qualifying-pathogens-under-the-food-and-drug-administration-safety-and). This designation seeks to incentivize new drug discovery and fast-track compounds for therapeutic intervention. Further, the Center for Disease Control and Prevention (CDC) provided descriptions of drug resistant bacteria and fluconazole resistant Candida (https://www.cdc.gov/drugresistance/pdf/ar-threats-2013-508.pdf).

Antifungal drug resistance is associated with one or more of the following mechanisms. Strains overexpress efflux pumps, such as Cdr1p, Cdr2p, Mdr1p (Candida Drug Resistance and Multiple Drug Resistant), have point mutations in the drug target protein such that the antifungal triazoles and caspofungin do not bind to fungal targets as well, or there is overexpression of target genes^2–5^. One of the rationales behind new drug discovery is to overcome the resistance to current antifungals such as the triazoles and echinocandins^4,6–8^. If so, then the sustained use of those drugs that select for resistant pathogens is possible, if synergy exists with another compound that counter selects for resistance. Recently, we demonstrated that a novel compound, bis[1,6-a:5′,6′-g]quinolizinium 8-methyl salt] (BQM), had broad activity against human pathogenic fungi^7^. The compound was especially active against MDR-resistant isolates of *Candida albicans*. Mechanistically, we noted that BQM only accumulated in *MDR1*-overexpressed and fluconazole-resistant clinical isolates of *Candida albicans* but not susceptible isolates, nor in an *mdr1*∆ and *mrr1*Δ null mutants^7^. Activated *MRR1* is a transcriptional regulator of resistance associated with *MDR1* overexpression^9^. Accumulation was correlated with increased susceptibility to BQM. By microarray, we also demonstrated an upregulation of many other transporters including those of the polyamine transporter family^7^. Susceptibility to BQM in MDR strains was reversed in the presence of polyamine transporter substrates as well as in a polyamine regulatory mutant.

We have recently utilized a compound library provided by the Developmental Therapeutics Program at the NIH/NCI (http://mli.nih.gov/mli/mlp-overview) to screen for inhibitors of pathogenic fungi. Many of the compounds from this library are known to have anti-cancer activity (or are active against other human diseases) and the mechanism of action of many has been suggested. These compounds are referred to as repurposed, if in fact additional activities (antifungal, for example) are identified^10,11^. In this regard, ~3000 compounds were screened for activity against a panel of pathogenic fungi representing several genera. Fluconazole-resistant isolates were among those screened. Of note, a thiosemicarbazone compound, NSC319726, revealed broad antifungal activity against a panel of pathogenic fungi including *Candida* species, *A. fumigatus*, and *C. neoformans* in the range of 0.1–2.0 µg/ml. Strikingly, NSC319726 was also highly inhibitory to multidrug-resistant isolates of *Candida* species. Importantly, no significant toxicity was found in wild type mice in previous studies^12^. As such, this manuscript highlights the susceptibility data and synergy of this compound. Also, we suggest a mechanism of action (MOA) of NSC319726, which entails inhibition of ribosome biogenesis and the induction of oxidative stress.

## Results

### NSC319726 has antifungal activity against a variety of pathogenic fungi

**Figure 1:**
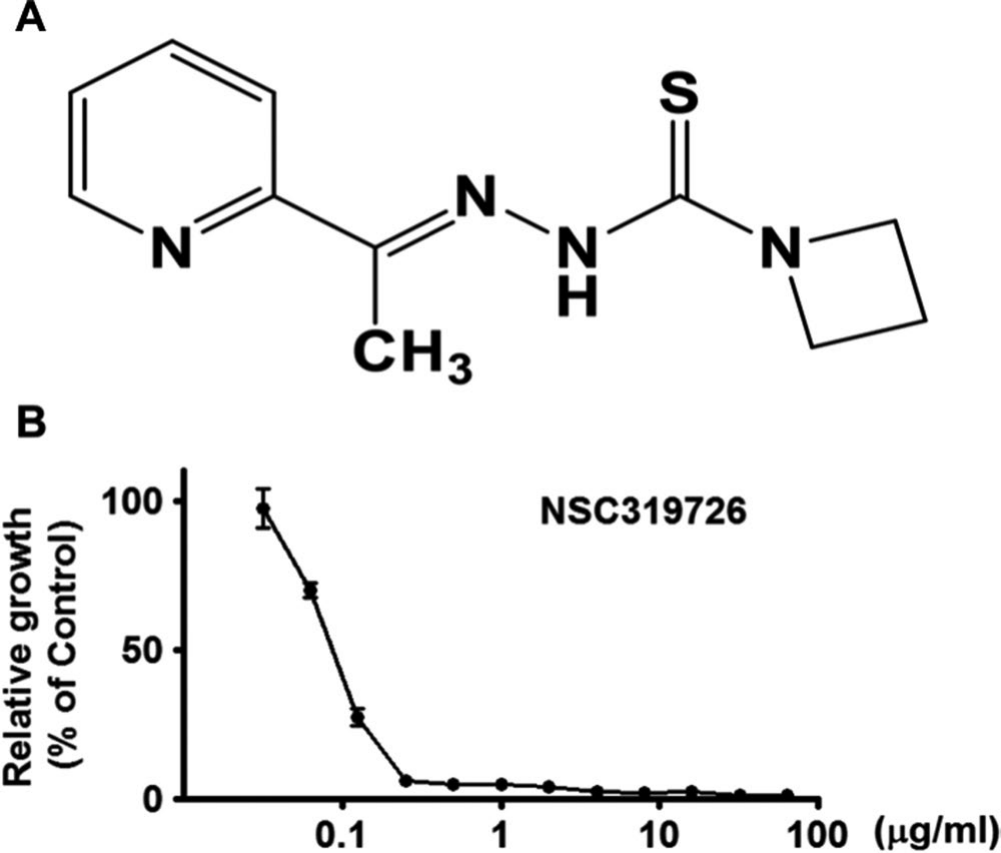
(**A**) The structure of NSC319726 is shown. (**B**) The relative growth of *C. albicans* (SC5314) is shown as a % of WT cell growth at concentrations of NSC319726 from 0.05–100 μg/mL compared to untreated cells.

The inhibitory activity of NSC319726 (Fig. 1A) at concentrations of 0–100 μg/mL was measured against *C. albicans* SC5314 (Fig. 1B). A 50% inhibition of growth was observed at a concentration of ~0.1 µg/mL of NSC319726. Susceptibilities to other *Candida* species, *Aspergillus fumigatus*, and isolates of *Cryptococcus neoformans* were also measured and compared to fluconazole **(**Table 1 **)**. Three of the *C. albicans* strains (5674, TW17, and G5) as well as *C. krusei* were resistant to fluconazole (32–128 μg/mL) but susceptible to NSC319726 at concentrations less than 1 μg/mL. All other isolates were susceptible to NSC319726 with MIC values near or lower than fluconazole.

**Table 1:** NSC319726 is inhibitory to isolates of *Candida* species as well as *Cryptococcus neoformans* and *Aspergillus fumigatus*. MIC assays were performed in a standard M27-A microdilution assay format. These data are compared to fluconazole. Three isolates of *C. albicans* and one of *C. krusei* were resistant to fluconazole but susceptible to NSC319726. MIC_50_ data are shown as μg/ml for each species.

Isolates/compounds	NCS319726	Fluconazole
*C. albicans (SC5314)*	0.1	0.25
*C. albicans (5674)*	0.4	128
*C. albicans (TW17)*	0.4	128
*C. albicans (G5)*	0.4	64
*C. parapsilosis*	0.1	1
*C. guilliermondii*	0.2	2
*C. glabrata*	0.2	2
*C. tropicalis*	0.2	0.5
*C. lusitaniae*	0.1	2
*C. apicola*	0.1	0.25
*C. krusei*	0.2	32
*Cr.neoformans H99*	0.2	4
*Cr.neoformans JEC-21*	0.1	2
*Aspergillus fumigatus*	2	16

The fungicidal activity of NSC319726 for *C. albicans* strains SC5314 and SN250 was also evaluated. By definition, the MFC/MIC ratio must be ≤4 for a compound to be defined as fungicidal. We demonstrate that NSC319726 is fungicidal to *C. albicans* strain SC5314 with an MFC/MIC = 1–2 for MIC_80_ or MIC_100_ values (Table 2).

**Table 2:** Fungicidal effects of NSC319726 (MFC/MIC) against *C. albicans* SC5314.

Inbt. or Killing percentage (%)	MIC (μg/ml)	MFC (μg/ml)	MFC/MIC
MIC_80_ or MFC_80_	0.125	0.25	2
MIC_100_ or MFC_100_	0.5	0.5	2

MFC/MIC ≤ 4 indicates a fungicidal effect.

### NSC319726 displays synergy with fluconazole, other triazoles and caspofungin

**Figure 2:**
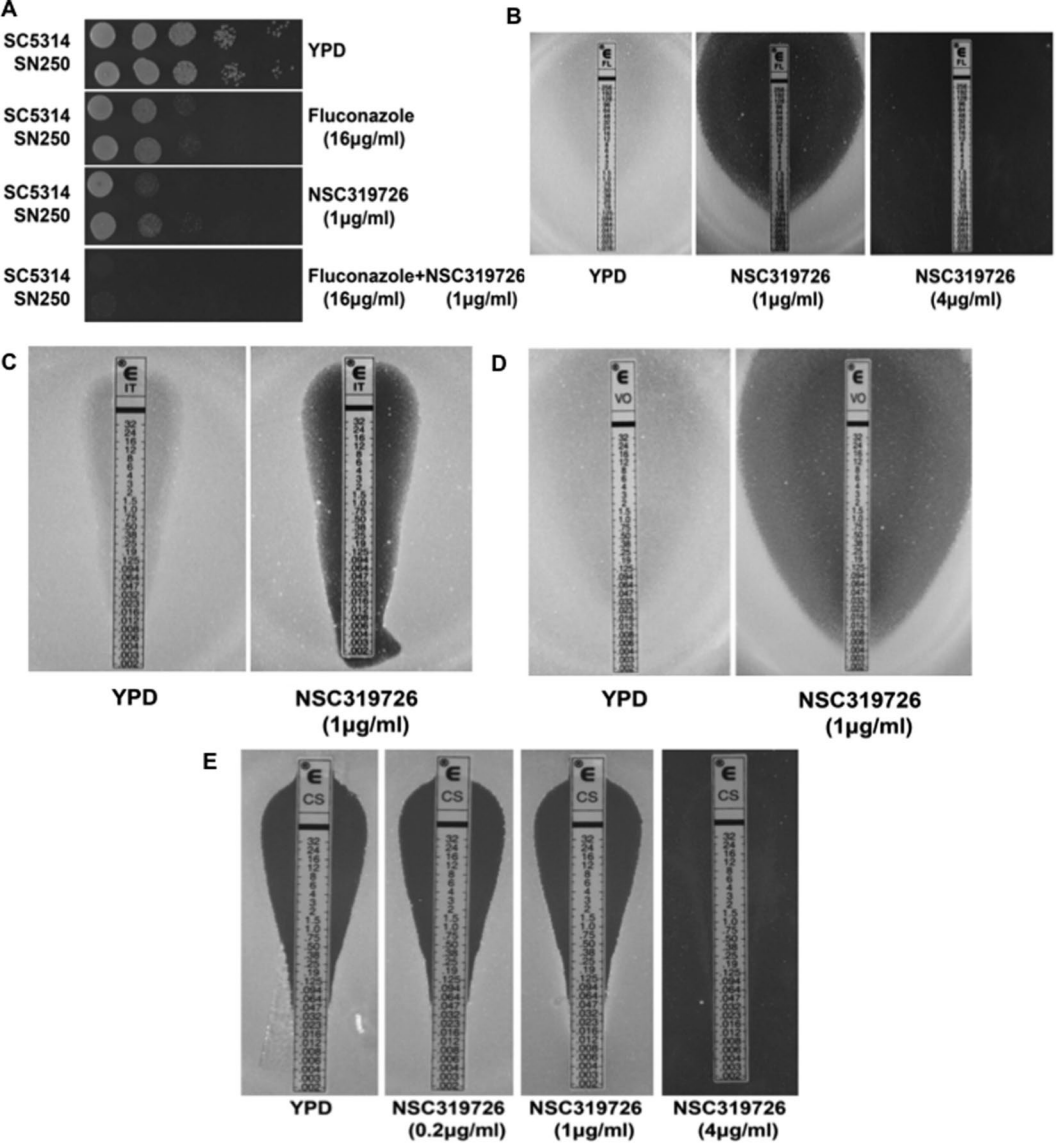
Synergy is demonstrated with NSC319726 and triazoles or caspofungin. (**A**) Drop plate assays are shown using NSC319726 alone (1 µg/mL), fluconazole alone (16 µg/mL), or NSC319726 in combination with fluconazole (FL) (16 µg/mL) (top to bottom panels). The combination of both drugs had a greater inhibition than either used alone. E-test assays were also performed with: (**B**) fluconazole (YPD only, far left) and NSC319726 at 1 μg (center) or 4 μg/ml; (**C**) itraconazole (IT) and NSC319726 (1 μg/mL); (**D**) or NSC319726 and voriconazole (VO), and (**E**) NSC319726 and caspofungin (CS) (0–4 μg/mL). Synergy is demonstrated for each combination, although more compound was needed to see maximum synergy with CS (4 μg/ml).

Initially, synergy was evaluated using drop plate assays **(**Fig. 2A). When used alone, both NSC319726 (1 μg/mL) and fluconazole (16 μg/mL) exhibited similar inhibitory activity against two strains of *C. albicans* (SC5314 and SN250) compared to growth of both strains without compounds (Fig. 2A, panels 1–3, top to bottom). However, NSC319726 and fluconazole together displayed synergy at the same concentrations as each drug added alone (Fig. 2A, bottom panel).

Further testing on synergy was done using E-tests (Epsilometer tests, Etests). We demonstrated that NSC319726 (in agar) caused synergy with fluconazole (Fl) (Fig. 2B), itraconazole (IT) (Fig. 2C), or voriconazole (VO) (Fig. 2D), each on E-test strips. Caspofungin (CS) (Fig. 2E) and NSC319726 were also synergistic but at a higher concentration of NSC319726 (Fig. 2E). Cultures were established to yield confluent growth on plates after incubation with E-test strips. The experiments demonstrate that NSC319726 synergizes with the triazoles but only at 4 μg/mL with caspofungin. Our reason for also choosing E-tests to measure synergy was the ease of handling these assays, the use of many more concentrations of triazoles and caspofungin, the 90% agreement of E-tests with broth dilution methods, and the simplicity of the assay.

### NSC319726 has minimal toxicity to human cell lines

Previously, NSC319726 was reported to inhibit growth of mammalian cancer cell lines with a p53 mutation^12^. P53 is a tumor suppressor protein. Healthy cells otherwise lacking the mutant p53 protein are not inhibited by NSC319726. Importantly, this compound was remarkably non-toxic to the wild type mouse strain Balb/c-3T3 and human WI38 fibroblast cells. Furthermore, no significant toxicity was found in wild type mice receiving 5 mg/kg/day^12^. We also observed minimal toxicity of NSC319726 *in vitro* against two human liver (hepatocyte) cell lines (HUH-7 and HepG-2) (Table 3). The IC_50_/MIC_50_ ratio (IC_50_, concentration of the compound that causes 50% cytotoxicity of liver cells; MIC_50_, minimum inhibitory concentration that causes 50% inhibition of fungal cell growth) was 800-fold higher following a 24 h incubation of cell lines with NSC319726 and 35–330-fold higher at 48 and 72 h. Therefore, NSC319726 is an attractive lead compound with strong antifungal activity and low toxicity to human cell lines in agreement with observations as stated above.

**Table 3:** Toxicity (IC_50_/MIC_50_) of NSC319726 to human liver cell lines Huh-7 and HepG-2 was measured at 24, 48, and 72 h post-treatment. The data are expressed as ratios of IC_50_/MIC_50_. Depending upon the time of incubation with the compound, the IC_50_ concentrations were 35–825-fold higher to achieve inhibition than the concentration to achieve an MIC_50_.

hours	Huh-7 Cells	HepG-2 Cells
24 h	825	780
48 h	330	330
72 h	35	35

Data are represented as an average of two experiments.

### NSC319726 affects ribosome biogenesis and reduces protein synthesis

**Figure 3:**
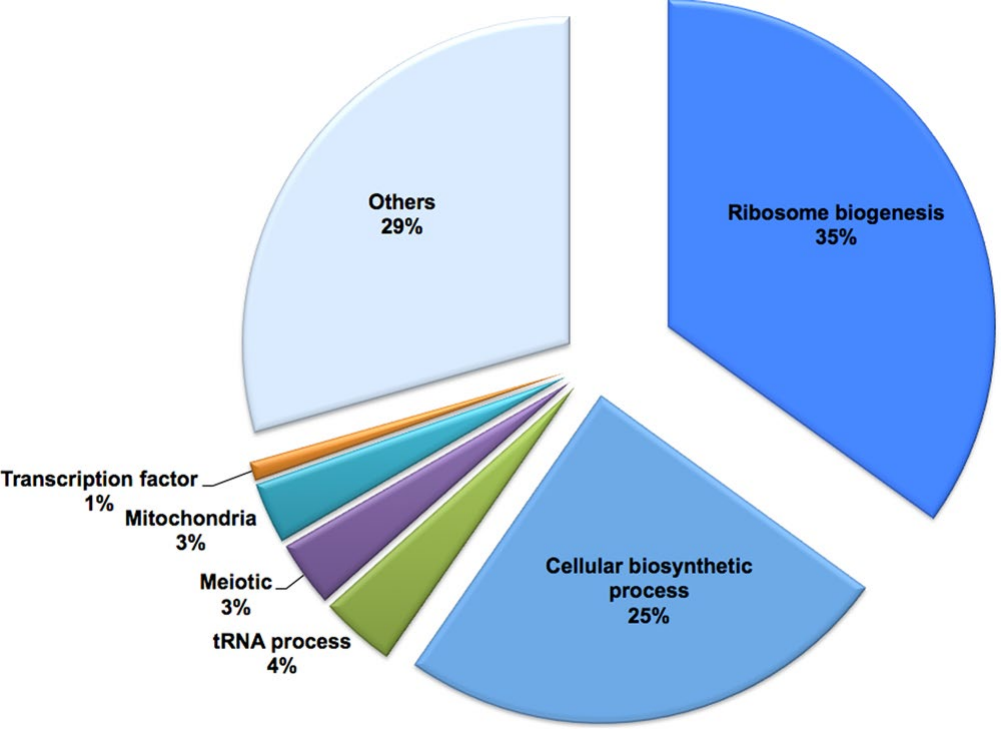
Microarray analysis of NSC319726-treated samples from *C. albicans* strain SC5314. Data are presented as a pie chart of functional gene categories (Gene Ontology Term analysis) of down regulated genes in treated cells compared to the untreated control. A total of 429 genes were down regulated, defined by a minimum 2-fold decrease of gene expression, including ribosome biogenesis genes (35%). Gene data in Figs 3 and 5 can be found in GSE-106486.

We next investigated the antifungal mechanism of action (MOA) of NSC319726 against *C. albicans* SC5314. We first used transcriptome analysis of wild type *C. albicans* treated or not with compound. Exponentially growing cells were treated for 60 min with 4 μg/ml of NSC319726. To identify the cellular pathways affected by treatment of *C. albicans*, we performed Gene Ontology (GO) analysis of the downregulated genes. Strikingly, the highest category of downregulated genes showed that they are associated with a large cluster of genes encoding components of the translational machinery in response to treatment (Fig. 3 and Supplemental Table 1). The vast majority of these genes had functions associated with ribosome biogenesis, including genes encoding cytoplasmic ribosomal proteins, rRNA processing enzymes, tRNA (transfer RNA) synthetases, RNA polymerase III subunits, as well as translation initiation and elongation factors. Ribosome biosynthesis is a highly complex and coordinated process. It is also one of the major metabolic pathways in all cells. Inhibition of ribosomal RNA metabolism has been shown to activate the signaling pathway for p53 induction^13^, and perturbation of ribosome biogenesis triggers a p53-activating signaling pathway independently of DNA damage^14^. p53 restoration by NSC319526 in mammalian cells is likely a subsequent effect due to modulation of ribosomal functions.

**Figure 4:**
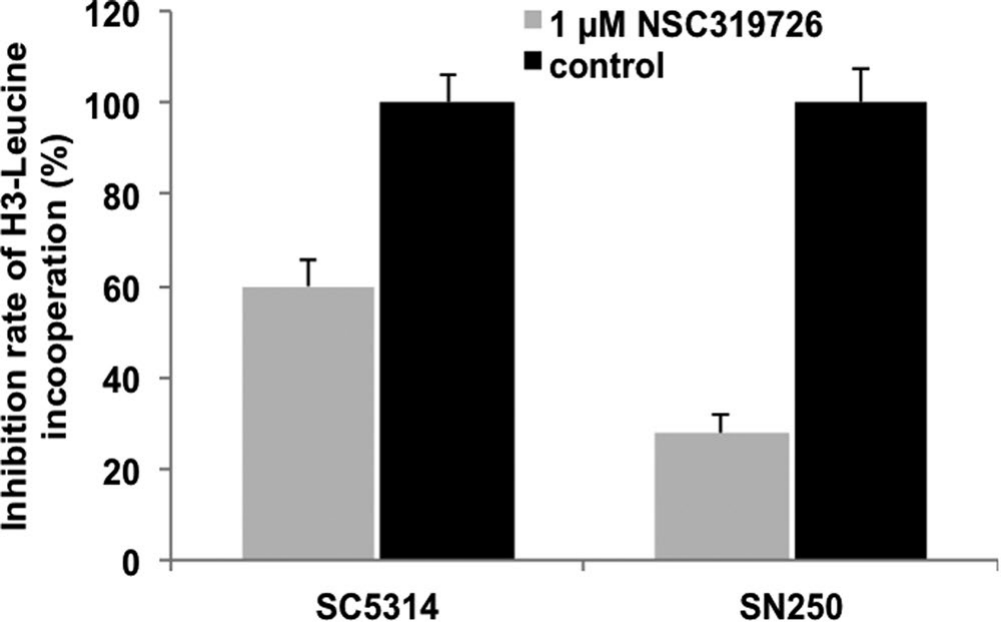
Protein synthesis (incorporation of ^3^H-leucine) is significantly reduced in NSC319726-treated compared to untreated cells of *C. albicans* strains SC5314 and SN250.

Since ribosome biogenesis and related genes are down regulated by NSC319726, we also measured protein synthesis in compound-treated and untreated cells. We show that protein synthesis (measured by incorporation of ^3^H-leucine) was reduced in treated fungal cells only (Fig. 4 **)**, although inhibition in treated *C. albicans* strains SC5314 and SN250 was strain-specific (40% and 75%, respectively).

**Figure 5:**
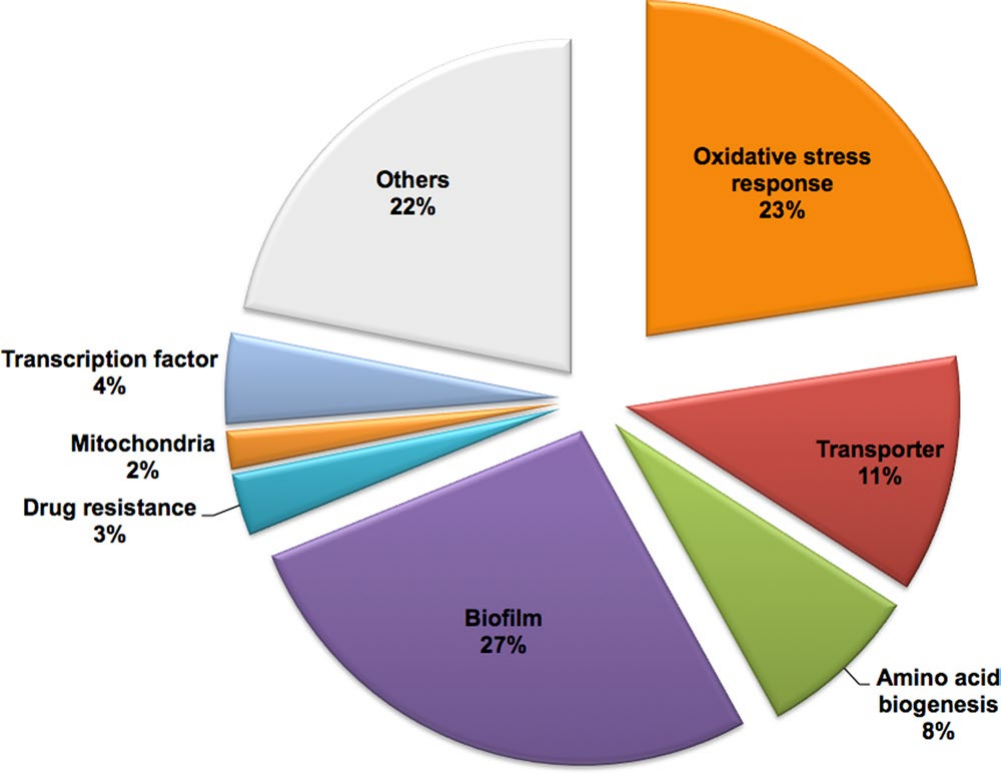
Microarray analysis of NSC319726-treated samples from *C. albicans* strain SC5314. Data are presented as a pie chart of functional gene categories (Gene Ontology Term analysis) of up regulated genes in treated cells compared to the untreated control. A total of 434 genes were up regulated, defined by a minimum 2-fold increase of gene expression, including core stress response genes (23%).

In addition to the ribosomal biogenesis pathway, we also note that genes associated with cellular biosynthetic process (25%), meiosis (3%), and mitochondria (3%) are downregulated in response to treatment (Fig. 3). Interestingly, core stress response genes were also found in abundance among the up regulated genes upon treatment, the majority of which are implicated in oxidative stress responses by their induction due to reactive oxygen species (ROS) (Fig. 5 and Supplemental Table 2), including genes encoding oxidoreductases (*EBP1*, *OYE32*, *PST2*), superoxide dismutases (*SOD2-4*), thioredoxin reductase (*TRR1*), glutathione reductase and peroxidase (*TTR1*, *orf19.86*), glutathione S-transferases (orf19.720, orf19.2613, orf19.3121, orf19.6947, orf19.2693) and an AP-1 family bZIP transcription factor, *CAP1*. The ability to sense and respond to ROS generated by the host immune system is required for *C. albicans* to survive in the human phagocytes^15,16^. Pharmacological ROS insults could have significant therapeutic implications to preferentially eliminate fungal pathogens. NSC319726 belongs to the thiosemicarbazone family, and a property of the thiosemicarbazones is that they modulate the redox state of the cells^17,18^.

**Figure 6:**
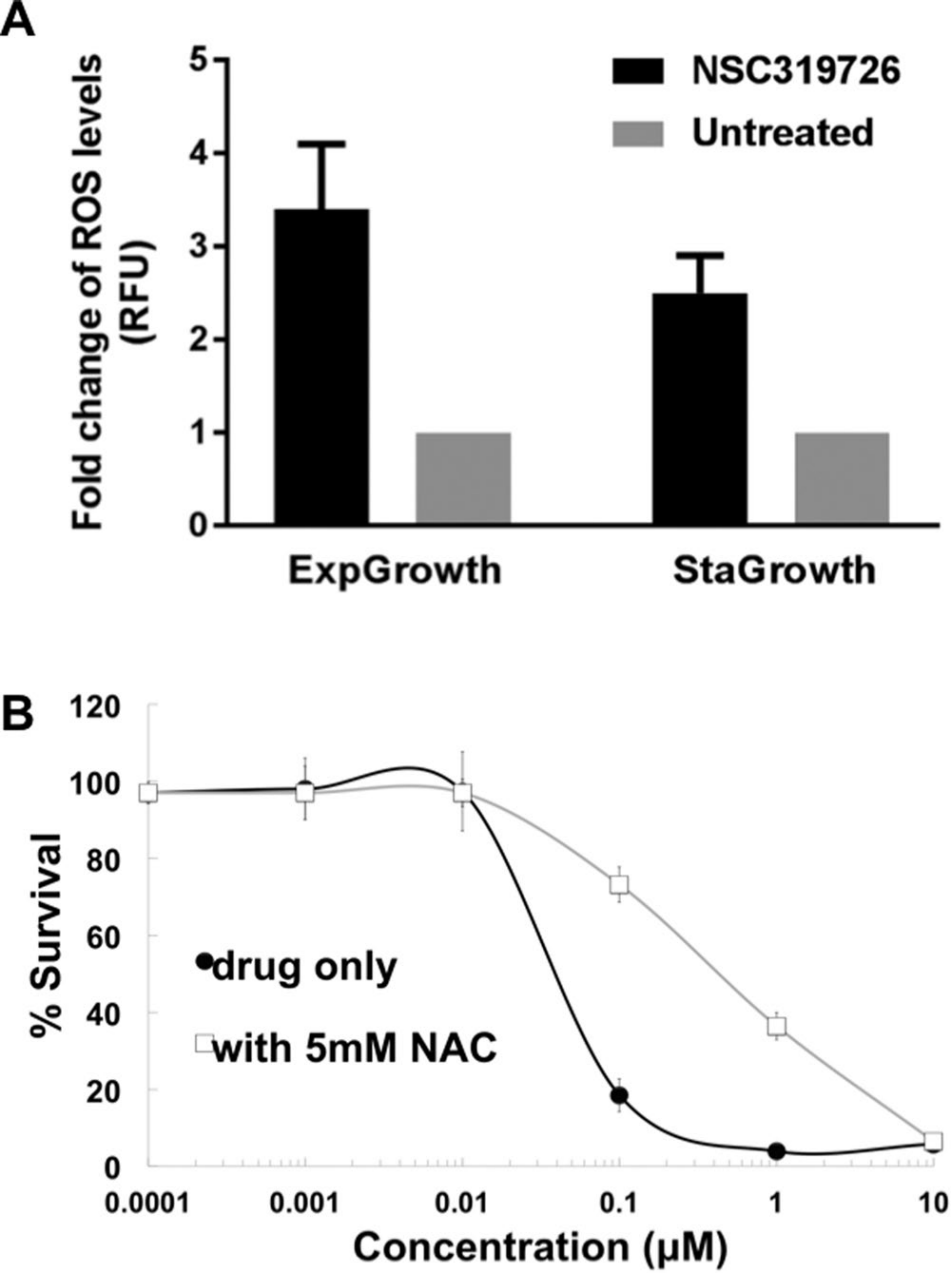
(**A**) ROS is increased in NSC19726-treated but not in untreated cells of *C. albicans* strain SC5314. ROS levels were higher from exponential phase growth compared to stationary phase growth. (**B**) The % survival of SC5314 in the presence of NSC19726 was increased when cells were co-treated with N-acetyl cysteine (NAC), which is an inhibitor of ROS.

In support of this, we found that NSC319726 caused an increase in ROS production in SC5314 *C. albicans* compared to untreated cells (Fig. 6A). ROS increases were noted in both exponential and stationary phase cells (Fig. 6A). The % survival of SC5314-treated cells was increased by the ROS scavenger N-acetyl cysteine (NAC) (Fig. 6B). Collectively, these data suggest that the antifungal mechanism of NSC319726 is at least partially related to toxic ROS production in *C. albicans*. What initiates the increase in ROS is unclear, although it may be related to the inhibitory effect of the compound on ribosome biogenesis and a core stress response that is unable to protect cells from stress damage.

Other pathway categories among the upregulated genes were functions associated with biofilm formation (27%), amino acid biogenesis (8%), mitochondrial functions (2%), transmembrane transporters (11%), and drug resistance (3%) (Fig. 5 and Supplemental Table 2). Induction of these genes might also be partially due to oxidative stress. Thus, a general stress response is caused by NSC319726. Down and upregulated gene data sets have been submitted to a public database (GSE-106486).

NSC319726 thus appears to cause numerous changes in cells at the transcriptional level as well as reducing cellular protein synthesis. As mentioned above, ROS levels contribute to cell death since reducing ROS with the NAC inhibitor increases viability. Thus, the primary effect of this compound that causes cell death is speculative.

## Discussion

Thiosemicarbazones such as NSC319726 have been evaluated for their antifungal and antibacterial activity^19–24^. Some of these reports are difficult to compare to our data since strains are often not identified, and MIC procedures do not always follow standardized methods. Soni *et al*., report activity against *Bacillus subtilis*, *E. coli*, *Streptomyces griseus*, *C. albicans*, and *Aspergillus niger* as determined by using a screening cup-plate assay^24^. Moderate activity was demonstrated by a series of derived thiosemicarbazones against *C. albicans* and non-albicans species^21^. A greater activity of specific thiosemicarbazones and hydrazones was noted against *Cladosporium cladosporioides* and *C. glabrata* but not *C. albicans* ^22^. While thiosemicarbazones were the focus of studies reported above, our data are clearly more expansive and include synergy and, preliminary MOA determinations.

Synergy of NSC319726 with triazoles was demonstrated although a higher concentration of NSC319726 was required to induce synergy with caspofungin. Synergy usually means each compound inhibits different targets. We suspect that NSC319726 targets the ribonucleoprotein complex, but high amounts of ROS are produced by treated cells, so the synergy may be due to ROS toxicity and ergosterol inhibition (triazole). As triazoles are known to select for resistance among isolates and species, compounds that synergize with triazoles may be useful in extending their shelf life.

We have used GO analysis to identify transcriptional changes associated with the treatment of *C. albicans*. In this regard, we demonstrate that NSC319726 causes a down regulation of an aggregate of genes encoding ribosomal biogenesis and related functions. Consequently, protein synthesis is reduced in treated cells as measured by leucine incorporation. Treated cells also have an increased ROS. Intriguingly, NSC319726 is one of two compounds that rescue the function of mutated TP53 (p53^R175H^)^12^. WT *TP53* encodes a tumor suppressor. However, the*TP53* mutation mentioned above results in a conformational change that decreases p53 binding to DNA and reduces transcription of *MDM2*, a known negative regulator of p53^12^. As a result of this mutation, tumorigenesis, invasion and metastasis occur.

As stated above, a direct effect of NSC319726 on ribosome biogenesis which causes cell death will require more study. In *Mycobacterium tuberculosis*, global transcriptional repression occurs during dormancy while low-abundant mRNAs are retained and are stable even under poor nutrient conditions^25^.

NSC319726, as mentioned above, has minimal toxicity to mice, and in this study, to human liver cell lines. We hypothesize that the compound binds to a closely related target in fungal cells. Unanswered is whether or not animals are protected against fungal infection. Those experiments along with *in vivo* toxicity determinations are needed. Repurposed compounds represent one of several approaches to the identification of new antifungals, reviewed in^26^.

## Materials and Methods

### Strains, maintenance and compounds

All strains used in the present study were maintained as frozen stocks and propagated on yeast extract-peptone-dextrose (YPD) agar when needed (1% yeast extract, 2% peptone, 2% glucose, 2% agar). All compounds were provided through the Developmental Therapeutics Program of the NIH/NCI, http://mli.nih.gov/mli/mlp-overview. Compounds included the 10 mM Diversity Set, Approved Oncology Drugs, 10 mM Natural Products Set, and 1 mM Mechanistic Set. Collectively, we screened 2,958 compounds. NSC319726 was chosen for this study because of its low MIC, presumed minimal toxicity, and the susceptibility of antifungal drug resistant strains.

### Antifungal susceptibility testing by broth microdilution

Drug susceptibility testing of all compounds was carried out in flat bottom, 96-well microtiter plates (Greiner Bio One) using the broth microdilution protocol according to the Clinical and Laboratory Standards Institute M-27A method. Growth inhibition of all strains was evaluated in the presence of varying concentrations of drug and reported as a % inhibition of untreated cells as previously described^6,7^. Experiments were repeated twice. Inhibition was measured based on OD_595_ growth data with or without compound (negative control). Compound screens for activity were determined against a panel of *Candida* species (fluconazole resistant or susceptible), *Aspergillus fumigatus*, and *Cryptococcus neoformans*.

### Minimal fungicidal concentration (MFC) determinations

The *in vitro* fungicidal activities were determined by sub-culturing 10 μl of the cell suspension from each microtiter plate well that showed complete inhibition in the MIC assays. Cells were cultured on YPD agar plates^27^. The cultures were incubated at 30 °C for 48 h. The MFC was determined to be the lowest drug concentration that resulted either in no growth or in fewer than five colonies per plate. If the ratio of the MFC to the MIC concentration was <4, the compound was designated as fungicidal; otherwise, a >4 ratio was designated as fungistatic.

### Antifungal susceptibility testing by drop plate assays and E-tests

The effects of NSC319726 on isolate susceptibility and resistance were also determined with E-test strips (AB Biodisk) on YPD medium. For *C. albicans*, cell densities of overnight cultures (YPD, 30 °C) were determined by hemocytometer counts. 5 × 10^5^ cells were plated to obtain confluent growth on YPD with or without compound (1–4 μg/ml) before application of antifungal test strips. Strips contained drugs (FL, fluconazole; IT, itraconazole; VO, voriconazole; CS, caspofungin) at concentrations ranging from 0.002 to 32 μg/mL. The plates were incubated at 35 °C, and evaluated at 24 and 48 h of incubation for growth inhibition. Growth inhibition was also visualized by plating 5 µL of ten-fold serial dilutions of cells onto YPD agar plates containing NSC319726 and/or fluconazole at the indicated concentrations. Cells had been grown overnight in YPD broth at 30 °C, washed with saline, and counted using a hemocytometer. Plates were photographed and evaluated at 48 h of incubation at 30 °C.

### Toxicity assays

Cell viability was determined by treating HepG-2 and Huh-7 human liver cells with NSC319726^27^. The 50% cell cytotoxicity (IC_50_) concentrations were calculated after 24, 48, and 72 hours by staining cells with neutral red according to standard procedures. Data are indicated as IC_50_ for NSC319726. We calculated IC_50_ /MIC_50_ ratios to determine the toxicity to cell lines expressed as fold-changes in toxicity.

### RNA preparation and microarray analysis

*C. albicans* SC5314 was grown at 30 °C overnight in YPD medium and washed twice with 0.1 M PBS (pH 7.0). Cells were suspended in RPMI 1640 medium and treated with NSC319726 (4 μg/ml) for one h. As previously described^7^, total RNA was extracted; the integrity and purity were assessed using an Agilent Bioanalyzer (Aglient Technologies) at OD_260/280_. One-color microarray-based gene expression analysis was done using the Agilent Low Input Quick Amp Labeling kit. *C. albicans* cDNA synthesis was carried out using 100 ng of total RNA, and all other methods followed the manufacturer’s instructions. Hybridization was carried out for 17 h in an Agilent SureHyb hybridization chamber, and the microarrays were scanned with an Agilent SCAN Microarray Scanner System using the AgilentHD_GX_1-color 5 μM protocol. A total of 6101 genes are represented by two sets of probes, both spotted in duplicate. Probes were randomly distributed. Tiff format image files were analyzed by Agilent Feature Extraction software. Cyanine 3 intensities were then logarithmetically transformed and statistically normalized. As previously described in our analysis, we adopted the cutoff for the parametric P value of <0.05 and fold change of >2 to determine the significant gene lists, using the false-discovery rate (FDR) as a reference. Genes that were up- or downregulated 2.0-fold were selected and considered to be differentially expressed. Gene ontology analysis was performed at the Candida genome database (CGD, www.candidagenome.org). The microarray data have been deposited to the GEO database with accession number [GSE106486].

### Protein Synthesis

TCA-precipitation of total cell protein: overnight growth of *C. albicans* wild type strains SC5314 and SN250 in YPD broth was diluted and grown to mid-log phase in 3-ml of Synthetic Complete (SC) medium (1 × 10^6^ cells/mL) containing 0.33 μM of leucine (final concentration) at 30 °C. The cells were collected, washed twice with PBS, and suspended in 1 ml of SC medium without leucine. Each cell suspension was divided into 2 parts, and one part was treated with 1 μM NSC319726 for 2 h at 30 °C, 200 rpm. The other half of the cell suspension was incubated at 30 °C for 2 h without drug treatment. Aliquots of 200 µL (1 × 10^6^ cells) were withdrawn in triplicate and incubated with 5 µCi L-[4,5-^3^H] of leucine for an additional 2 h at 30 °C with gentle shaking. Non-incorporated leucine was removed by washing cells with PBS twice (pH 7.0), and cells were incubated with 800 µL of 5% trichloroacetic acid, then heated at 90 °C for 20 min. For all samples, radiolabelled protein was collected on nitrocellulose filters (0.45 µM, Millipore) and rinsed with water and ethanol 3 times. The radioactivity of each dried filter (in triplicate) was measured using a scintillation counter. ^3^H-Leucine incorporation of each sample was determined by comparing the cpm of drug treated samples versus untreated cells. Experiments were repeated twice.

### Measurements of cell reactive oxidant species (ROS) in treated cells

As previously described^28^, intracellular ROS production was detected by staining cells with the ROS-sensitive fluorescent dye DCFH-DA (2,7-dichlorofluoresceindiacetate; Sigma). Cells from 25-mL cultures grown at 30 °C overnight in YPD medium were collected and washed twice with phosphate buffered saline, pH 7.0 (PBS). The pellets were suspended to 10^6^ cells in 10 ml of PBS plus 2% glucose and treated with or without 10 μM DCFH-DA for 30 min at 37 °C in the dark. Cells from each sample were collected, washed twice with PBS after staining, and suspended in PBS plus 2% glucose, then treated with NSC319726 at the indicated concentrations for 2 h at 30 °C. The fluorescent intensity was measured using a FACScan flow cytometer (Becton Dickinson). Propidium iodide (PI) was added to each sample to measure dead cells prior to DCFH-DA assays. The mean fluorescence of ROS was quantified only in live cells. ROS production by cells was measured during exponential or stationary phase growth with or without NSC319726.

### Statistical analysis

The Student t test was used for all analyses. Differences were considered significant when P was < 0.05.

## Electronic supplementary material

Supplementary Information S1

Supplementary Information S2
